# *In vitro* small molecule screening to inform novel candidates for use in fluconazole combination therapy *in vivo* against *Coccidioides*

**DOI:** 10.1128/spectrum.01008-24

**Published:** 2024-08-20

**Authors:** Heather L. Mead, Michael Valentine, Holly Yin, George R. Thompson III, Paul Keim, David M. Engelthaler, Bridget M. Barker

**Affiliations:** 1 The Translational Genomics Research Institute North, Flagstaff, Arizona, USA; 2 Beckman Research Institute of the City of Hope, Duarte, California, USA; 3 Department of Internal Medicine, Division of Infectious Diseases, University of California-Davis, Sacramento, California, USA; 4 Department of Medical Microbiology and Immunology, University of California-Davis, Sacramento, California, USA; 5 Department of Biological Sciences, The Pathogen and Microbiome Institute, Northern Arizona University, Flagstaff, Arizona, USA; Geisel School of Medicine at Dartmouth, Lebanon, New Hampshire, USA

**Keywords:** coccidioidomycosis, Valley Fever, antifungal therapy, fungal infection, antifungal therapy, adjunctive agent, *Coccidioides*, sertraline

## Abstract

Identifying improved treatments for severe and refractory coccidioidomycosis (Valley fever) is needed. This endemic fungal disease is common in North and South America, and cases have increased substantially over the last 30 years. The current standard of care, oral daily fluconazole, often fails to completely eradicate *Coccidioides* infection; however, the high cost of identifying new compounds effective in treating Valley fever is a barrier to improving treatment. Therefore, repurposing existing pharmaceutical agents in combination with fluconazole therapy is an attractive option. We screened the Library of Pharmacologically Active Compounds (LOPAC) small molecule library for compounds that inhibited fungal growth *in vitro* and determined IC_50_ values for a subset of compounds. Based on these findings, we tested a small subset of these agents to validate the screen, as well as to test the performance of fluconazole in a combination therapy approach, as compared with fluconazole alone, in a murine model. We observed that combination therapy of tamoxifen:fluconazole and sertraline:fluconazole significantly reduced the burden of live fungus in the lung compared with fluconazole alone, and we observed reduced or nonexistent dissemination. These results suggest that tamoxifen and sertraline may be repurposed as adjunctive agents in the treatment of this important fungal disease.

**IMPORTANCE:**

Developing new drugs, especially for regional orphan diseases, such as Valley Fever, is a slow and costly endeavor. However, there is a wealth of FDA-approved drugs available for repurposing, offering a more economical and expedited approach to improve treatment. Those existing compounds with antifungal properties can become novel therapies with relative ease: a considerable advantage for patients in need of alternative treatment. Despite the scope of remaining tasks, our comprehensive screening of potential candidates has revealed promising combinations for further exploration. This effort outlines a practical pipeline for Valley fever drug screening and identifies viable drug combinations that could impact patients more rapidly than single drug development pathways.

## INTRODUCTION

*Coccidioides* spp. are pathogenic environmental fungi responsible for considerable morbidity and mortality within the United States (1). These organisms cause the disease coccidioidomycosis (Valley Fever [VF]). Clinical manifestations vary depending upon both the extent of infection and the immune status of the host. Although many infections are of subclinical severity, some patients develop flu-like symptoms (e.g., cough, fever, chills, headaches, joint pain, rash) consistent with respiratory infection, whereas others develop extrathoracic dissemination requiring long courses of antifungal treatment. Patients with severe coccidioidomycosis often are treated with an amphotericin B formulation, which carries significant potential nephrotoxicity and is currently available only intravenously. Those with mild to moderate infection are treated with triazole antifungals (fluconazole, itraconazole, voriconazole, posaconazole, or isavuconazole) (2, 3). These agents have exhibited efficacy against *Coccidioides* in both animal and human studies; however, these agents may have toxic effects, considerable drug–drug interactions exist, and refractory disease may persist despite treatment, mandating the continued search for additional therapeutic options (4).

In contrast to traditional drug development pathways, the repurposing (or repositioning) of old drugs for new indications (such as antifungals) may significantly reduce the time, effort, and costs required to bring new agents to clinical trials (5). The Prestwick Chemical library (LOPAC) is commercially available and includes 1,280 drugs approved by the Food and Drug Administration (FDA), which enables high throughput in *vitro* screening for biological activity. Recent work has shown that repurposing is a viable strategy, with synergistic *in vitro* activity leading to clinical trials evaluating the efficacy of combination therapy in cryptococcal infection (6).

Currently, no published work has investigated the feasibility of using existing FDA-approved drugs (LOPAC) in combination with triazoles in the treatment of VF. Therefore, we measured the ability of 1,280 compounds from the LOPAC small molecule library to inhibit *Coccidioides* growth *in vitro* (U.S. Patent 11,446,353) and determined IC_50_ for a subset of compounds with inhibition properties. From these *in vitro* findings, we identified that three compounds, which could be administered orally, were likely to have acceptable mammalian toxicity profiles, and based on other studies (7–10), they may demonstrate antifungal behavior or adjunctive potential when combined with fluconazole, the recommended first-line of treatment for VF. In summary, we report the findings from small molecule screening and evaluate the efficacy of these compounds in a murine model of infection.

## MATERIALS AND METHODS

### Fungal growth for small molecule screening

*Coccidioides posadasii* ∆*cts2*/∆*ard1*/∆*cts3* (NR166, BEI Resources) was grown for 5–8 days at 30°C on a shaking incubator in 1% glucose 0.5% yeast extract (1xGYE) liquid media containing 50 µg/mL hygromycin B, then filtered using sterile Miracloth. One milliliter of this filtered culture was seeded in 100 mL of fresh 1xGYE and grown for another 5–6 days under the same conditions. Before compound screening, cultures were diluted 1:1 in fresh 1xGYE media, and 40 µL of the diluted culture was added to experimental wells of a 384-well plate using a Multidrop liquid dispenser (ThermoFisher).

### LOPAC compound screening

Using the ATS-100 Acoustic Transfer System (EDC Biosystems), experimental wells were dosed with tested compounds at final concentrations of 1 µM and 5 µM; wells without test compounds were inoculated with an equivalent volume of vehicle (DMSO). Each 384-well plate contained the following controls: wells containing media only, wells containing strain NR-166 *Coccidioides,* and wells containing strain NR-166 *Coccidioides* treated with staurosporine as a positive control. Whole control plates of media only and strain NR-166 *Coccidioides* in DMSO were also included in the study. After compound dosing was complete, the OD600 was measured using an Envision Multi-mode plate reader (PerkinElmer). Plates were sealed to prevent evaporation and incubated at 30°C. Successive OD600 measurements were taken at 24-h intervals until 120 h by using an Envision Multi-mode reader. This compound screening procedure was performed, in totality, twice.

### Dose–response curve

A subset of LOPAC compounds of interest was first prepared to a stock concentration of 20 mM in DMSO, and then serially diluted threefold in DMSO to produce another 11 concentrations ranging from 20 to 11.3 µM. Using the ATS-100, the serially diluted compound stocks and DMSO vehicle control were added (100 nL) to untreated *Coccidioides* wells in a 1:1 ratio, producing an effective 65-test concentration range of 50 to 28 nM of each compound. A final DMSO concentration of 0.25% was maintained throughout the assay plate, rendering potential DMSO toxicity an experimental constant and eliminating the necessity of accounting for it on a well-by-well basis. Optical density readings at 600 nm (OD600) were taken immediately after the addition of the compounds and repeated every 24 h for 168 h. The experiment was repeated twice.

### *In vivo* assays

All vertebrate animal work was conducted with Northern Arizona University IACUC approval (#16–009). Infection was established using 1,000 arthroconidia suspended in sterile PBS from *C. posadasii* strain Silveira (ATCC 28868) instilled intranasally under anesthesia with ketamine/xylazine (80 mg/kg/8 mg/kg) injected intraperitoneally. Fungal cultures were grown, harvested, and quantified as previously described (11). CD1 male (*n* = 10 per treatment group, *n* = 4 PBS, and *n* = 5 infected) mice between the ages of 4–6 weeks were given tamoxifen (Sigma) in peanut oil, administered via oral gavage, for 3 days (12) before fungal infection at 200 mg/kg/day (13) or sertraline hydrochloride (Aurobindo 15 mg/kg/day) in saline, via oral gavage, for 6 days (14, 15) before fungal infection at 15 mg/kg/day. From 24-h post-infection, animals in the treatment groups were dosed, via oral gavage every 24 h with tamoxifen citrate (Sigma, 200 mg/kg/day), sertraline hydrochloride (Aurobindo, 15 mg/kg/day), fluconazole suspension (Diflucan, 15 mg/kg/day), vanoxerine (Sigma, 10 mg/kg/day); or combinations of tamoxifen:fluconazole (15:200 mg/kg/day), sertraline:fluconazole (15:15 mg/kg/day), or vanoxerine:fluconazole (10:15 mg/kg/day). Table S3 outlines experimental groups and details group numbers. Mice were euthanized 6–8 days post-infection, and lungs and spleens were harvested. Post-necropsy fungal burden was determined by homogenizing the entire lung or spleen, respectively, in 1 mL of sterile PBS. Serial dilutions of each homogenate were plated in duplicate on 2xGYE (2% glucose, 1% yeast agar) and incubated for 3–4 days at 30°C. Colony-forming units (CFU) were counted and averaged between dilutions and replicates as described (11). Plates were retained for 2 weeks to observe for possible delayed growth.

### Statistical analysis

Statistical analysis and graphical representations for *in vitro* work were conducted using Excel. Statistical analysis and graphical representations for *in vivo* work were conducted using RStudio software R version 4.2.1 (16). Several model fits and model parameters were tested [(linear regression using residual squared error, observing residual patterns) and (Poisson and negative binomial using deviance, residual deviance, and AIC)] to determine the goodness of fit for the data. A negative binomial regression model was selected based on these criteria (MASS package (17) and used to determine the reduction in log fungal burden between treatment groups. Due to extreme weight loss, the tamoxifen-treated animals had to be terminated on day six instead of the planned day eight. Only one fluconazole-treated mouse was sacrificed on the same day for fungal burden comparison. The remaining fluconazole-treated mice were culled on day eight. All fluconazole mice were used in the binomial model.

## RESULTS

### LOPAC compound screening

Screening of 1,280 LOPAC compounds was completed for two concentrations of compounds, 1 µM and 5 µM, and the experiment was performed twice. In the control plates and control wells, no change in the OD600 was detected in the media-only wells over the course of the experiment. The 48-h data point was the first time point where fungal growth was detectable compared with media-only controls. The difference between OD600 signal and media control wells increased over the course of the experiment. Assay performance was assessed by Z-factor (a measure of statistical effect size), which improved over time (0.5–0.9) and coefficient of variation, which ranged between 5% and 14%. (Figures S1-S5). In the first experiment, at either 5 µM or 1 µM, 42 of the compounds tested inhibited fungal growth by 50% or better (Table S1). In the second experiment, 56 of the compounds tested inhibited fungal growth by >50% at 5 µM or 1 µM (Table S1). Of the compounds tested here, 36 were shared between tests, whereas seven were unique in test one and 19 in test two.

### Dose–response curve

A subset of 46 compounds that inhibited fungal growth by >50% in runs one or two were serially diluted and used to determine IC_50_ values. A serial dilution of staurosporine was used as a positive control, and the IC_50_ values of staurosporine remained generally consistent over the course of the 120-h experiment (Fig. S6). IC_50_ values for the compounds tested are listed in Table S2. These results, a literature search evaluating potential mammalian toxicity of the evaluated compounds, and available pharmacokinetic data-informed selection and dosing strategies of the *in vivo* work. We began our work by testing tamoxifen, vanoxerine, and sertraline in combination with fluconazole, a first-line antifungal used in the treatment of coccidioidomycosis.

### Tamoxifen:fluconazole treatment decreases fungal burden but contributes to weight loss

**Fig 1:**
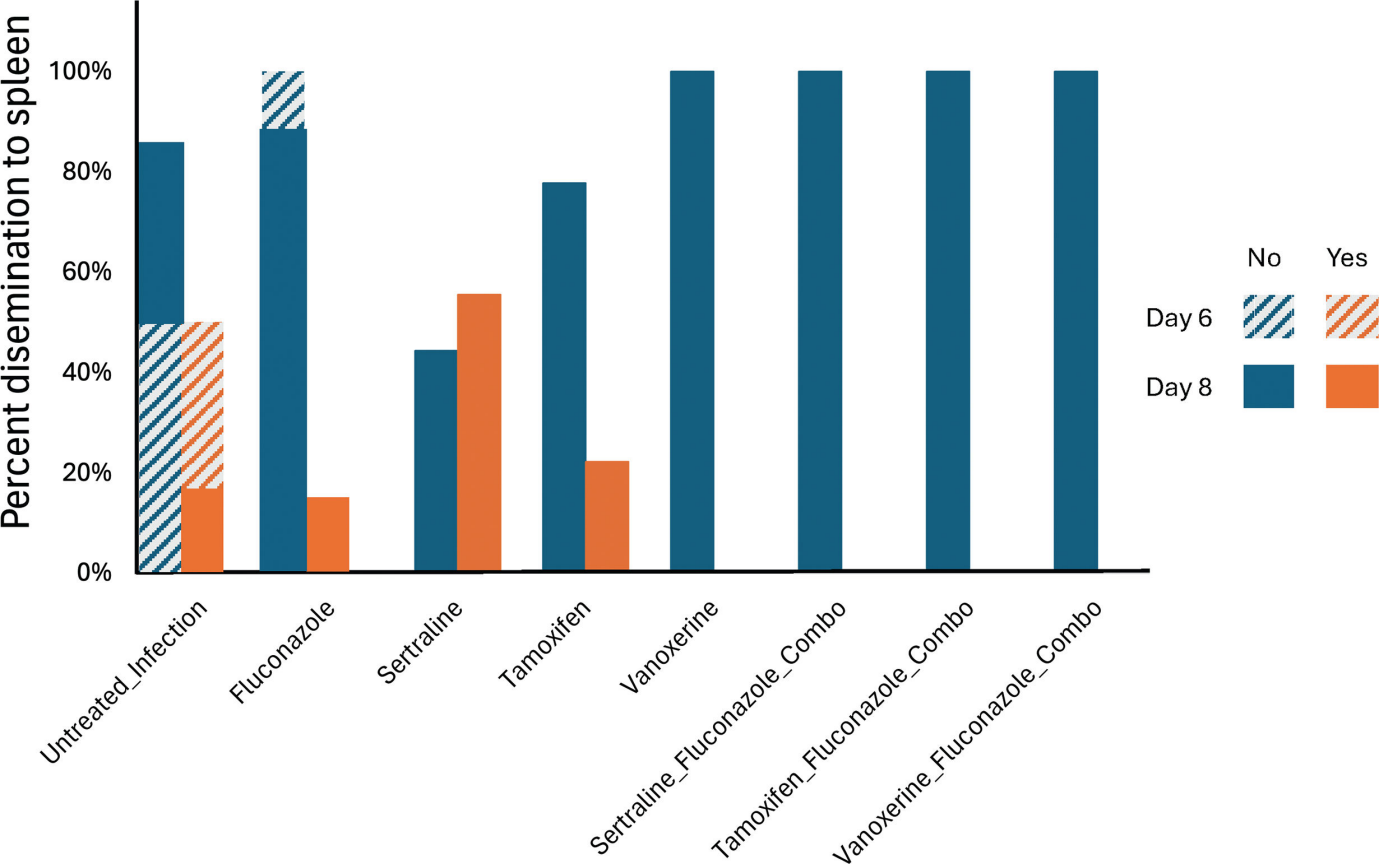
The observed dissemination to spleen for each treatment group. Dissemination (no, blue) did not occur in any combination therapy groups, but dissemination did occur (yes, orange) in a subset of untreated infection, fluconazole-only, sertraline-only, and tamoxifen-only mice. Tamoxifen therapy mice and a subset of controls were culled on day 6 (patterned lines), and all other mice were terminated on day 8 (no pattern).

**Fig 2:**
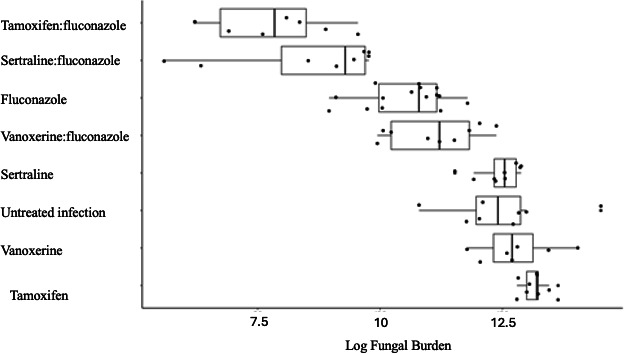
Combination of tamoxifen:fluconazole and sertraline:fluconazole therapy significantly reduces fungal burden compared with fluconazole alone (*P* < 0.001). A negative binomial was used to compare the weighted average fungal burden in the lungs for each treatment group.

The planned terminal date for each experiment was 8 days post-infection. However, all mice treated with either tamoxifen alone or in combination with fluconazole experienced a loss of >20% of total body weight and were therefore euthanized on day 6. Therefore, a subset of mice from the untreated infection (*n* = 2), fluconazole treatment (*n* = 1), and control mice (*n* = 2) were also euthanized on day 6 instead of day 8 for statistical comparison. Fungal burden in the lung was measured as the average CFU grown from whole lung homogenates in 1 mL of PBS. On day 6, the untreated infection group had 1 × 10^6^ (SD 1.2 × 10^6^ cells/mL), fluconazole alone 7 × 10^3^ cells/mL (*N* = 1, no SD), tamoxifen alone 5.3 × 10^5^ (SD 1.5 × 10^5^ cells/mL), and tamoxifen:fluconazole 4 × 10^3^ (SD 4.6 × 10^3^ cells/mL) total fungal colonies. The small number of control mice available limited statistical inference, so we used day 6 and day 8 mice to build the negative binomial model, given that we do not expect an exponential increase in growth for this slow-growing fungus. In this model, the combination therapy was observed to significantly reduce the log fungal burden in the lung compared with fluconazole alone (log reduction −2.46, *P* < 0.001, Table 1; Fig. 2). We observed dissemination to the spleen in two groups of mice culled on day 6: 50% untreated infected, and 22% of tamoxifen-treated mice. No dissemination was observed in the combination therapy group (Fig. 1). Uninfected mice gained weight (median, IQR; 1.55 g, 0.75) over the duration of treatment, whereas untreated infected mice lost weight (median, IQR; −2.65 g, 0.85). Tamoxifen:fluconazole-treated mice lost weight during infection (median, IQR; −1.7 g, 2.3) as described above; one fluconazole mouse was sacrificed on day 6 (gained 1 g); and the remaining fluconazole+infection mice were sacrificed on day 8 (median, IQR; −0.6 g, 1.05).

**TABLE 1:** Log lung fungal burden between combination groups compared with fluconazole alone

Treatment group	Fungal burden reduced	Log count difference^*a*^	*P*-value
Fluconazole:sertraline	Yes	−1.5838	3.68 × 10^−06^
Fluconazole:tamoxifen	Yes	−2.4593	6.66 × 10^−13^
Fluconazole:vanoxerine	No	0.6742	0.0408

^*a*^ Negative binomial regression dispersion parameter 1.637 (theta), AIC 1817.7.

### Vanoxerine:fluconazole treatment is less effective than fluconazole alone

Mice were sacrificed on day 8. The average fungal burden for each group was untreated infection (2.5 × 10^5^) (SD 1.5 × 10^5^), fluconazole alone (4.6 × 10^4^) (SD 3.4 × 10^4^), vanoxerine alone (4.6 × 10^5^) (SD 4.0 × 10^5^), and vanoxerine:fluconazole (9.3 × 10^4^) (SD 7.4 × 10^4^) total fungal colonies. Applying a negative binomial model, we discerned that combination therapy significantly increased the log fungal burden in the murine lung as compared with treatment with fluconazole alone (log increase 0.67, *P* < 0.001, Table 1; Fig. 2). Dissemination in the spleen was observed in the untreated infected group (16%) and the fluconazole group (14%). Dissemination was not observed in the vanoxerine alone or combination therapy group (Fig. 1). Uninfected mice lost minimal weight during the treatment time (median, IQR; −0.3 g, 1.56), whereas untreated infected mice lost weight (median, IQR; −4.1 g, 2.3) for fluconazole alone (median, IQR; −0.6 g, 1.05), vanoxerine alone (median, IQR; −3.7 g, 4.5), vanoxerine:fluconazole (median, IQR; −1.7 g, 2.3).

### Sertraline:fluconazole treatment decreases fungal burden without impacting weight

Mice treated were euthanized on day 8. The average lung fungal burden for each group was untreated infection (2.5 × 10^5^) (SD 1.5 × 10^5^), fluconazole alone (4.6 × 10^4^) (SD 3.4 × 10^4^), sertraline alone (2.7 × 10^5^) (SD 1.0 × 10^5^), and sertraline:fluconazole (9.8 × 10^3^) (SD 7.2 × 10^3^) total fungal colonies. Using a negative binomial model, we determined that combination therapy significantly reduced the log fungal burden in the lung when compared with fluconazole alone (log reduction −1.58, *P* < 0.001, Table 1; Fig. 2). Dissemination in the spleen was observed in three groups: untreated infected (16%), fluconazole alone (14%), and sertraline alone but not in the combination therapy group (55%) (Fig. 1). Healthy mice maintained weight during the treatment time (median, IQR; 0–0.3 g, 1.56), whereas untreated infected mice lost weight (median, IQR; −4.1 g, 2.3). Fluconazole alone (median, IQR; −0.6 g, 1.05) and sertraline:fluconazole groups gained weight (median, IQR; 0.5 g, 1.1).

## DISCUSSION

Coccidioidomycosis remains a common infectious agent within endemic regions of the Americas. Chronic or disseminated forms of the disease carry high associated morbidity, and in some cases may be fatal (18, 19). Treatment durations range from weeks to months to life-long therapy; and shorter courses of therapy, or alternative treatment regimens in patients that are intolerant or refractory to current antifungals, are urgently needed.

The repurposing of currently available non-antifungal agents as adjunctive agents for coccidioidomycosis is a potential strategy to support available treatment options. In our screening, we noted good concordance between both LOPAC screens at each respective concentration. Growth inhibition of >50% at any time point and concentration in both screens was used to select compounds for further investigation. Using this criterion, 36 compounds were prioritized as hits from the LOPAC library screen, representing a hit rate of 2.7%. The IC_50_ dose–response indicated several compounds were effective at low concentrations, supporting that this method is an effective way to interrogate *Coccidioides* growth. We evaluated three of these agents (e.g., tamoxifen, vanoxerine, and sertraline) in a murine model based on availability of an oral formulation of the drug in question, the ability to achieve meaningful serum and tissue concentrations in a rodent model, and available toxicity data associated with systemic administration.

Tamoxifen has anti-estrogenic activity and is used primarily in the treatment of early and advanced breast cancer, but off-target effects are common (8). Tamoxifen possesses antifungal properties, as first noted against *Saccharomyces cerevisiae* (9). Subsequent work has shown tamoxifen disrupts yeast cell integrity and has antifungal activity against *S. cerevisiae* and *Cryptococcus* (8, 10). The addition of tamoxifen to fluconazole treatment regimens resulted in fungicidal activity against *Cryptococcus* spp. *in vitro* (20) and in a murine model of infection (10), and human trials began shortly thereafter (6). This open-label, randomized-control trial failed to demonstrate an increased rate of cryptococcal clearance from the cerebrospinal fluid of patients receiving tamoxifen+fluconazole and amphotericin combination therapy. However, tamoxifen remains a potentially attractive agent in the treatment of VF because of the clear effects of estrogen and sex hormones on *Coccidioides* infections (21, 22). Tamoxifen concentrates in brain tissue (10- to 100-fold compared with plasma) and macrophage phagosomes; and it is off-patent, potentially decreasing drug costs (6, 23, 24).

*In vivo*, tamoxifen:fluconazole treatments performed better than the current standard of care, fluconazole, (*P* < 0.001) by log reduction of colony-forming units (CFUs) in the lung. Additionally, there was no dissemination to the spleen in the combination treatment group; however, this was not significantly different from fluconazole or tamoxifen treatment alone. Interestingly, although tamoxifen treatment alone did not decrease fungal burden in the lung, we did see some evidence of protection in the spleen: only one tamoxifen-treated mouse displayed any fungal growth in the spleen, suggesting possible inhibition of dissemination. However, tamoxifen-treated mice were sacrificed early due to weight loss, clearly associated with treatment, exceeding 10% of starting body weight. In the control treatment groups, where mice received either tamoxifen alone or in combination with fluconazole, the mice also lost significant weight: an average of 11%. Tamoxifen interferes with glucose and lipid metabolism in mice (25), which is almost certainly the cause of weight loss.

Vanoxerine is a dopamine reuptake inhibitor and antiarrhythmic medication previously evaluated in several clinical trials for these purposes (26–28). Prior attempts to evaluate vanoxerine as an antimicrobial agent revealed antimycobacterial effects (7). Disruption of the mycobacterial membrane, loss of membrane electrical potential, and the prevention of electrolyte efflux have been proposed as major mechanisms of vanoxerine’s antimycobacterial activity (29), and these same mechanisms may be responsible for its antifungal properties.

In our murine studies evaluating drug efficacy, vanoxerine:fluconazole combination therapy resulted in increased lung fungal burdens compared to fluconazole treatment alone. Although statistically significant, the *P*-value was just below the cutoff threshold (*P*-value = 0.0408). Dissemination was not seen in the combination group, suggesting that the addition of vanoxerine may have prevented extrapulmonary dissemination, yet increased the burden of pulmonary disease. The reasons for this are unclear.

Sertraline is a selective serotonin reuptake inhibitor (SSRI) used in the treatment of depression and anxiety disorders, among other conditions. Potent *in vitro* and *in vivo* fungicidal activities have been demonstrated against *Cryptococcus* spp., and the major mechanism of antifungal activity has been postulated as dose-dependent inhibition of eukaryotic translational initiation factor 3 (Tif3) by sertraline, with a resultant decrease in protein synthesis (15, 30). Based on these pre-clinical findings, a prospective, open-label, dose-finding pilot study was undertaken; and those receiving sertraline were found to have more rapid CSF cryptococcal clearance and a lower incidence of immune reconstitution inflammatory syndrome and relapse than was observed by similar cohorts in prior studies (31). Seemingly contradicting this, a phase 3 study of HIV-infected patients with cryptococcal meningitis was disappointing, with no reduction in mortality or rate of fungal clearance from patient cerebrospinal fluid reported in those receiving adjunctive sertraline compared to placebo (32). However, all patients in this trial received amphotericin B deoxycholate, and it is possible that its rapid fungicidal effects obviated any additive effects of sertraline. An alternative explanation might be that the time to the steady-state of sertraline (7–14 days) was too long, for this brief study period, for a benefit to become apparent. In contrast, the treatment of severe forms of VF requires weeks to months of therapy, and is lifelong in some cases. A delay in steady-state achievement of an adjunctive treatment agent is thus not detrimental and may still prove a useful addition.

In our murine model of infection, using a similar dosing strategy to that used in established models of cryptococcal infection, combination sertraline:fluconazole treatments performed better than fluconazole (*P* < 0.001), as determined by measuring CFU in the lung. Additionally, there was no dissemination to the spleen in the combination treatment group; however, this was not significantly different from fluconazole treatment alone. Interestingly, sertraline treatment alone did not provide any protection: in both lung and spleen, similar burdens of infection were observed. All mice survived until the final day of the experiment. Weights were similar among all treatment groups, and all were higher than untreated mice, as expected.

In summary, we investigated the potential of three pharmaceutical agents, tamoxifen, vanoxerine, and sertraline to potentiate the fungistatic ability of fluconazole. We observed that combination therapy with either tamoxifen:fluconazole or sertraline:fluconazole reduced fungal burden in a murine model when compared with fluconazole alone. However, our findings are limited because mice metabolize tamoxifen differently than humans, which necessitated early euthanasia, but there was a significant decrease in fungal burden. In contrast, sertraline:fluconazole-treated mice responded positively to treatment and had reduced fungal burden. These observations support the further development of combination therapy specifically for the treatment of coccidioidal meningitis. Future investigations for additional *in vivo* screening of small molecules in Table S2 that fit our original selection criteria could increase the number of combination therapies. Unfortunately, some compounds are prohibitively expensive or difficult to work with in current formulations. Thus, future work could determine the mode of action of these targets, and support the subsequent development of specific effective analogs and molecular targets. Our findings are limited to *in vitro* screening and within a murine model. Although these systems do not always predict human outcomes, they are useful tools in the development of new therapeutics.

## SUPPLEMENTAL MATERIAL

Supplemental figures spectrum.01008-24-s0001.pdf Fig. S1 to S6.

Supplemental tables spectrum.01008-24-s0002.docx Tables S1 to S3.

ASM does not own the copyrights to Supplemental Material that may be linked to, or accessed through, an article. The authors have granted ASM a non-exclusive, world-wide license to publish the Supplemental Material files. Please contact the corresponding author directly for reuse.
