# Supplementary material for: In vitro small molecule screening to inform novel candidates for use in fluconazole combination therapy in vivo against Coccidioides
Source: Microbiol Spectr. 2024 Aug 20;12(10):e01008-24. doi: 10.1128/spectrum.01008-24 (PMC11448266; doi:10.1128/spectrum.01008-24)
Supplement: Supplemental figures — Fig. S1 to S6. [file spectrum.01008-24-s0001.pdf]

1A

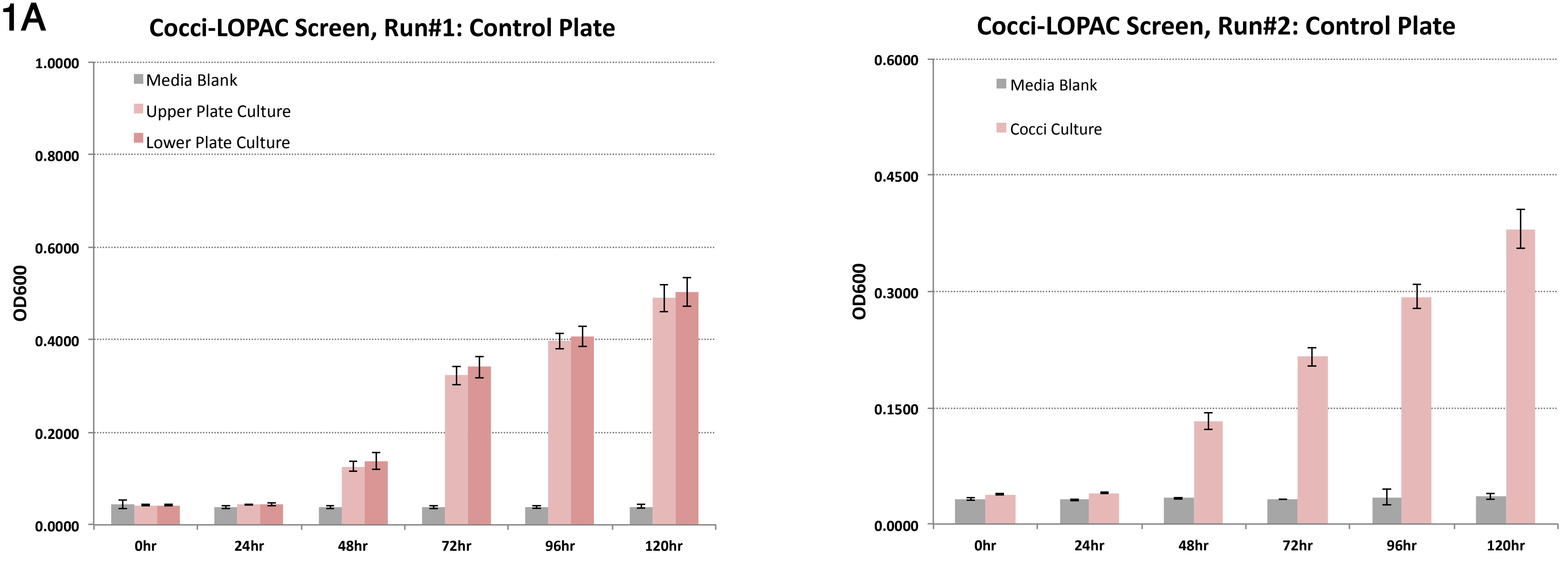

1B

run 1

Cocci-LOPAC Control		0hr	24hr	48hr	72hr	96hr	120hr
Blank	Avg OD600	0.0436	0.0381	0.0383	0.0385	0.0389	0.0394
	%CV	22.4%	9.1%	8.5%	9.1%	8.5%	8.9%
Upper Plate Culture	Avg OD600	0.0420	0.0440	0.1256	0.3230	0.3974	0.4901
	%CV	**3.0%**	**3.3%**	**8.8%**	**6.2%**	**4.3%**	**6.1%**
	OD600/Blank	1.0	1.2	3.3	8.4	10.2	12.4
	Z Factor	-23.9653	-1.4370	**0.5104**	**0.7513**	**0.8300**	**0.7781**
Lower Plate Culture	Avg OD600	0.0421	0.0437	0.1378	0.3408	0.4072	0.5026
	%CV	**3.6%**	**5.8%**	12.9%	**6.8%**	**5.4%**	**6.1%**
	OD600/Blank	1.0	1.1	3.6	8.8	10.5	12.7
		**Z Factor**	-26.4470	-2.1382	0.3694	**0.7351**	**0.70778**

run 2

Cocci-LOPAC Control		0hr	24hr	48hr	72hr	96hr	120hr
Blank	Avg OD600	0.0322	0.0314	0.0334	0.0321	0.0348	0.0355
	%CV	3.3%	2.0%	2.9%	2.4%	7.6%	9.8%
Cocci Culture	Avg OD600	0.0386	0.0405	0.1326	0.2155	0.2932	0.3803
	%CV	**2.5%**	**4.5%**	**8.8%**	**5.6%**	**6.1%**	**7.6%**
	OD600/Blank	1.2	1.3	3.8	6.5	8.2	10.5
	Z Factor	0.0387	0.1735	**0.6263**	**0.7913**	**0.7741**	**0.7316**

Supplemental figure 1. Assay control plates. A) Coccidioides growth is detected beginning at 48 hours. B) Average OD 600, coefficient of variation (CV) and Z factor for both runs.

2A

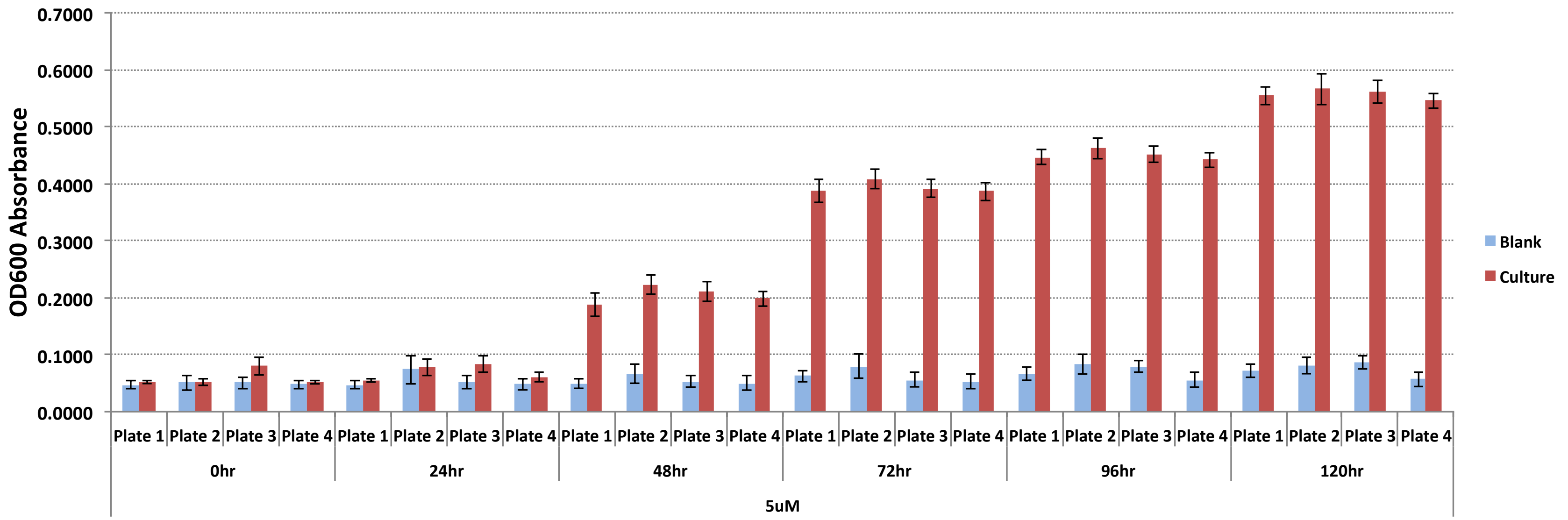

2B

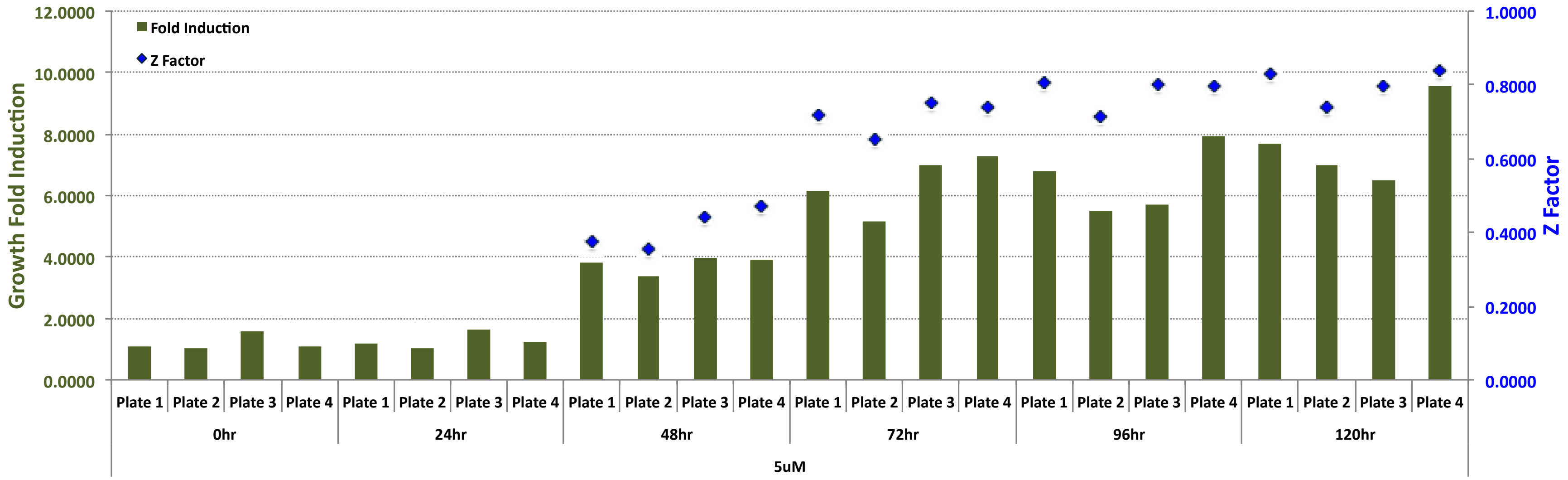

Supplemental Figure 2. Controls from LOPAC 5uM Run 1. A) OD values of wells containing Coccidioides in DMSO or media alone during 120 hour time series. B) Average OD 600, coefficient of variation (CV) and Z factor for run 1.

3A

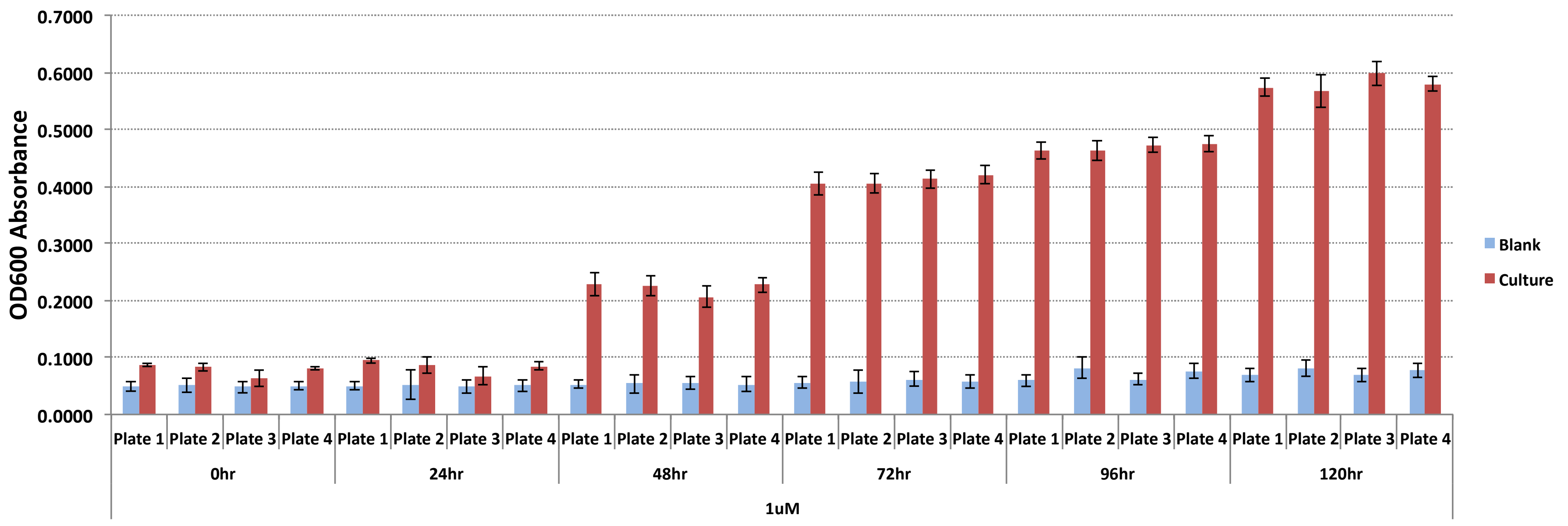

3B

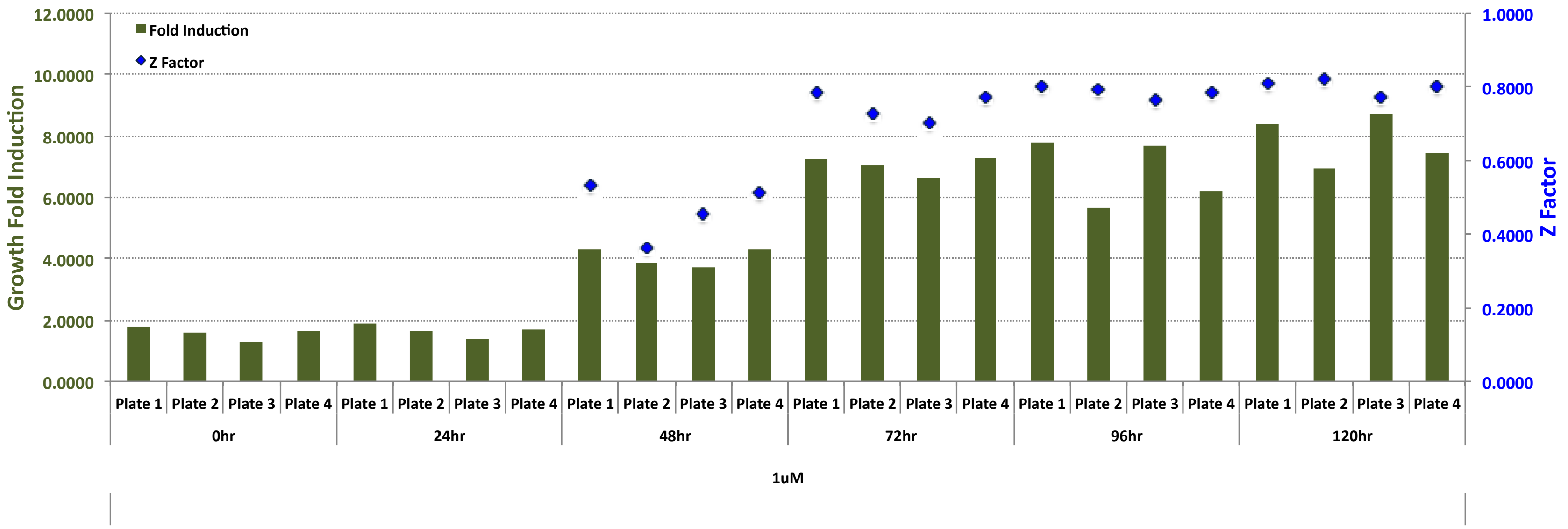

Supplemental Figure 3. Controls from LOPAC 1 uM Run 1. A) OD values of wells containing Coccidioides in DMSO or media alone during 120 hour time series. B) Average OD 600, coefficient of variation (CV) and Z factor for run 1.

4A

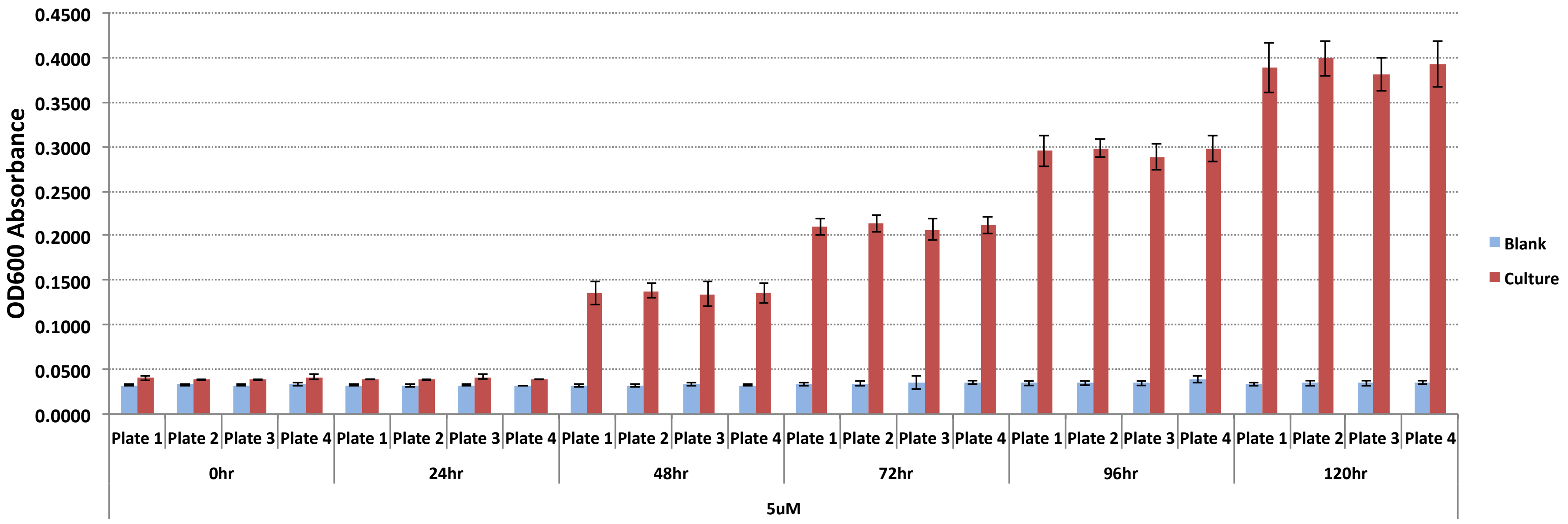

4B

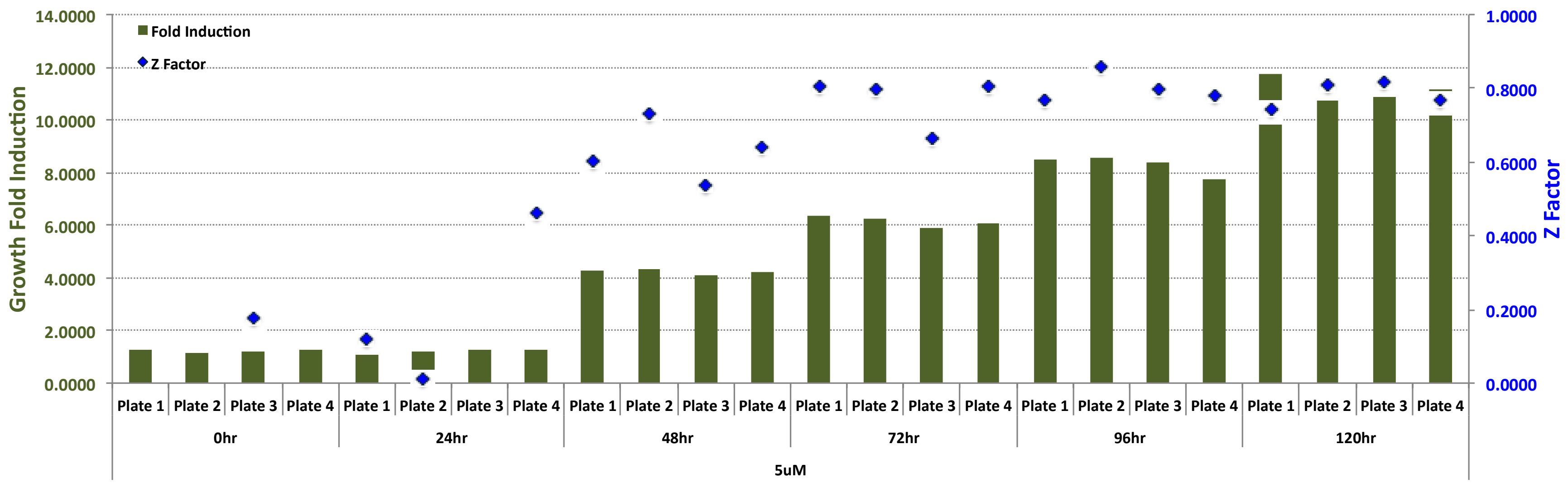

Supplemental Figure 4. Controls from LOPAC 5uM Run 2. A) OD values of wells containing Coccidioides in DMSO or media alone during 120 hour time series. B) Average OD 600, coefficient of variation (CV) and Z factor for run 1.

5A

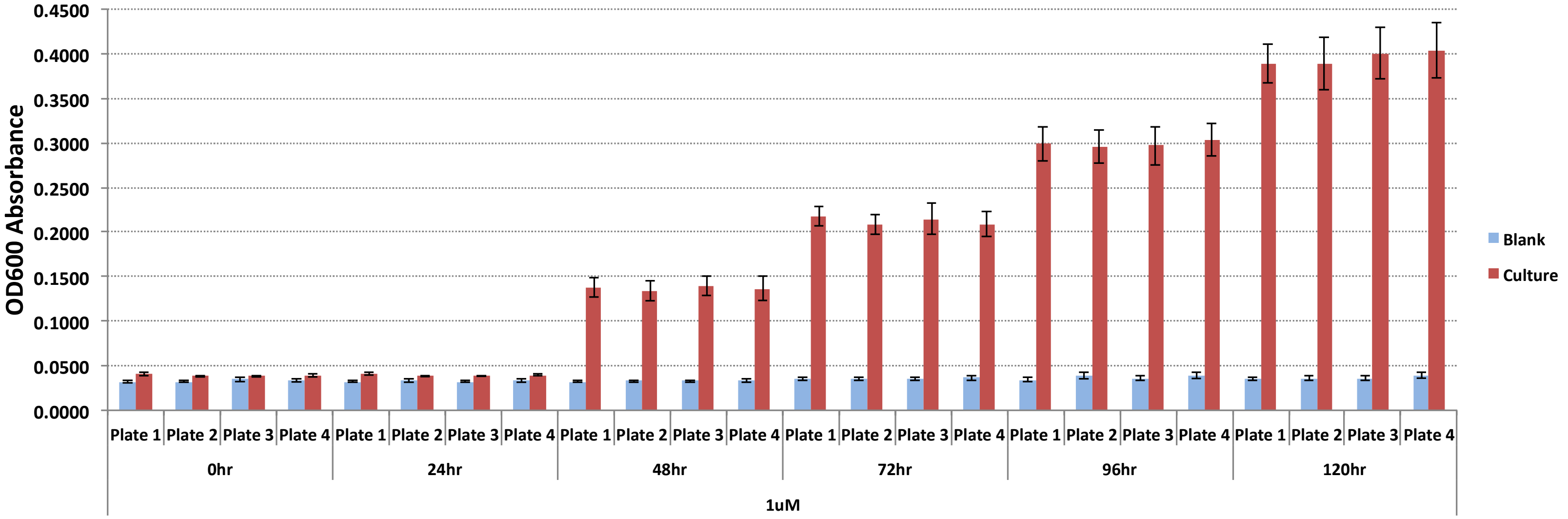

5B

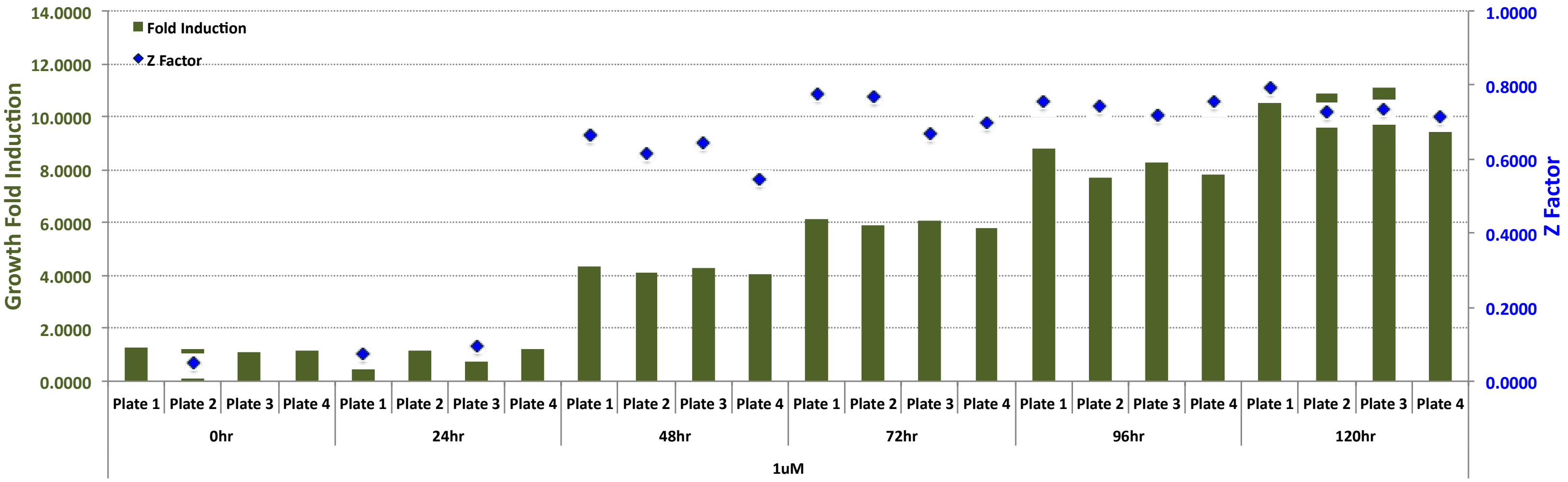

Supplemental Figure 5. Controls from LOPAC 1 uM Run 2. A) OD values of wells containing Coccidioides in DMSO or media alone during 120 hour time series. B) Average OD 600, coefficient of variation (CV) and Z factor for run 1.

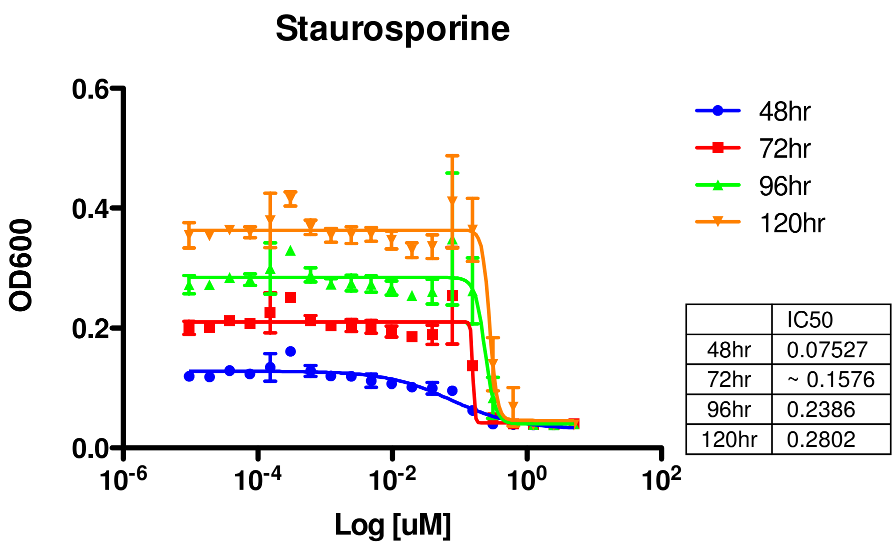

	IC50
48hr	0.07527
72hr	~ 0.1576
96hr	0.2386
120hr	0.2802

Supplemental Figure 6. IC50 values of Coccidioides only well with Staurosprine
