# Supplementary material for: In vitro small molecule screening to inform novel candidates for use in fluconazole combination therapy in vivo against Coccidioides
Source: Microbiol Spectr. 2024 Aug 20;12(10):e01008-24. doi: 10.1128/spectrum.01008-24 (PMC11448266; doi:10.1128/spectrum.01008-24)
Supplement: Supplemental tables — Tables S1 to S3. [file spectrum.01008-24-s0002.docx]

| Supplemental Table 1 | |
| --- | --- |
| Run 1 | Run 2 |
| **AC-93253 iodide** | (±)-Octoclothepin maleate |
| **AS 604850** | 1,3 ,5-tris(4-hydroxyphenyl)-4-propyl-1 H-pyrazole |
| **Auranofin** | **AC-93253 iodide** |
| Bay 11-7082 | Artemether |
| **Bay 11-7085** | **AS 604850** |
| **beta-Lapachone** | **Auranofin** |
| **BIO** | **Bay 11-7085** |
| **Calcimycin** | **beta-Lapachone** |
| **Calmidazolium chloride** | **BIO** |
| **CGP 57380** | **Calcimycin** |
| **Chelerythrine chloride** | **Calmidazolium chloride** |
| **Clotrimazole** | **CGP 57380** |
| **Cyclosporin A** | CGS-12066A maleate |
| **Dequalinium chloride hydrate** | **Chelerythrine chloride** |
| **Eliprodil** | Chlorpromazine hydrochloride |
| **Ellipticine** | Chlorprothixene hydrochloride |
| Fluphenazine dihydrochloride | cis-(Z)-Flupenthixol dihydrochloride |
| **GBR-12909 dihydrochloride** | **Clotrimazole** |
| **GBR-12935 dihydrochloride** | **Cyclosporin A** |
| **IMS2186** | **Dequalinium chloride hydrate** |
| **Indirubin-3' -oxime** | **Eliprodil** |
| **Ketoconazole** | **Ellipticine** |
| Niclosamide | **GBR-12909 dihydrochloride** |
| **Nocodazole** | **GBR-12935 dihydrochloride** |
| **PD-166285 hydrate** | Ifenprodil tartrate |
| **PD173952** | **IMS2186** |
| **Pentarnidine isethionate** | Indatraline hydrochloride |
| Rotenone | **Indirubin-3'-oxime** |
| **Sanguinarine chloride** | JS-K |
| **Sertraline hydrochloride** | **Ketoconazole** |
| **SR 59230A oxalate** | LP 12 hydrochloride hydrate |
| Stattic | Methiothepin mesylate |
| **SU 5416** | ML-7 |
| **Tarnoxifen** | **Nocodazole** |
| **Tarnoxifen citrate** | **PD-166285 hydrate** |
| **Trifluoperazine dihydrochloride** | **PD173952** |
| **Triflupromazine hydrochloride** | **Pentarnidine isethionate** |
| **Tyrphostin A9** | Prochlorperazine dimaleate |
| Tyrphostin AG 879 | Quinacrine dihydrochloride |
| U-73343 | Ritanserin |
| **Voriconazole** | Ruthenium red |
| **ZM 39923 hydrochloride** | **Sanguinarine chloride** |
|  | SCH 58261 |
|  | **Sertraline hydrochloride** |
|  | **SR 59230A oxalate** |
|  | **SU 5416** |
|  | **Tarnoxifen** |
|  | **Tarnoxifen citrate** |
|  | TBBz |
|  | Thioridazine hydrochloride |
|  | **Trifluoperazine dihydrochloride** |
|  | **Triflupromazine hydrochloride** |
|  | **Tyrphostin A9** |
|  | **Voriconazole** |
|  | Ziprasidone hydrochloride monohydrate |
|  | **ZM 39923 hydrochloride** |

Supplemental Table 1. List of compounds tested which inhibited fungal growth by >50% at 5µM or 1µM in run 1 or run 2. Bold names inhibited fungal growth by >50% in both experiments.

| **Supplemental Table 2. IC50 Values** | | | | | | | | |
| --- | --- | --- | --- | --- | --- | --- | --- | --- |
| **Plate** | **Compound** | **Experiment** | **IC50 Value [uM]** | | | | | |
|  |  |  | **48hr** | **72hr** | **96hr** | **120hr** | **144hr** | **168hr** |
| Assay Plate 1 | Clotrimazole | Run#1 | 0.070 | 0.096 | 0.102 | 0.087 | 0.065 | 0.069 |
|  |  | Run#2 | 0.037 | 0.032 | 0.059 | 0.048 | 0.050 | 0.065 |
|  | Dequalinium Chloride Hydrate | Run#1 | 1.438 | 1.347 | 1.618 | 1.687 | 1.535 | 1.611 |
|  |  | Run#2 | 0.836 | 0.873 | 0.962 | 0.936 | 0.956 | 0.977 |
|  | Thioridazine hydrochloride | Run#1 | 23.570 | 21.860 | 15.030 | 12.960 | 10.590 | 11.240 |
|  |  | Run#2 | 29.670 | 17.360 | 17.790 | 16.790 | 16.160 | 15.950 |
|  | Ketoconazole | Run#1 | 0.078 | 0.108 | 0.109 | 0.149 | 0.066 | 0.070 |
|  |  | Run#2 | 0.042 | 0.034 | 0.077 | 0.065 | 0.047 | 0.077 |
|  | Trifluoperazine dihydrochlorid | Run#1 | 18.290 | 20.110 | 14.430 | 12.720 | 11.320 | 12.020 |
|  |  | Run#2 | 27.370 | 19.870 | 20.620 | 17.010 | 16.300 | 16.230 |
|  | Fluphenazine dihydrochloride | Run#1 | 23.810 | 23.300 | 17.450 | 16.430 | 14.500 | 14.130 |
|  |  | Run#2 | 28.460 | 20.640 | 24.170 | 19.570 | 16.420 | 16.670 |
|  | Ruthenium Red | Run#1 | >50 | >50 | >50 | >50 | >50 | >50 |
|  |  | Run#2 | >50 | >50 | >50 | >50 | >50 | >50 |
|  | Niclosamide | Run#1 | 0.817 | 1.258 | 1.593 | 2.049 | 1.549 | 1.546 |
|  |  | Run#2 | 0.235 | 0.349 | 0.771 | 0.611 | 0.627 | 0.759 |
|  | Tamoxifen | Run#1 | 3.079 | 3.598 | 2.397 | 2.377 | 1.348 | 1.004 |
|  |  | Run#2 | 1.430 | 0.880 | 1.853 | 1.606 | 1.079 | 1.348 |
|  | Tamoxifen citrate salt | Run#1 | 2.820 | 3.502 | 2.337 | 2.306 | 1.378 | 0.991 |
|  |  | Run#2 | 1.091 | 0.773 | 1.771 | 1.495 | 1.075 | 1.347 |
|  | Pentamidine isethionate salt | Run#1 | 39.340 | 39.650 | 27.660 | 32.480 | 20.240 | 17.660 |
|  |  | Run#2 | 42.880 | 38.630 | 45.440 | 38.240 | 31.030 | 40.580 |
|  | GBR 12909 dihydrochloride | Run#1 | 22.420 | 22.050 | 14.580 | 12.240 | 7.184 | 6.229 |
|  |  | Run#2 | 14.930 | 9.769 | 12.360 | 9.728 | 7.057 | 9.232 |
|  | Octoclothepin maleate salt | Run#1 | 27.720 | 26.450 | 15.480 | 14.070 | 11.380 | 11.300 |
|  |  | Run#2 | 30.530 | 18.080 | 21.550 | 18.680 | 14.990 | 16.210 |
|  | ZM 39923 | Run#1 | 8.406 | 20.340 | 24.610 | 28.460 | 23.630 | 25.990 |
|  |  | Run#2 | 3.006 | 4.468 | 8.575 | 7.512 | 7.784 | 12.520 |
|  | Cyclosporin A | Run#1 | 1.482 | 1.841 | 0.977 | 1.748 | 0.743 | 0.725 |
|  |  | Run#2 | 0.992 | 1.231 | 0.633 | 1.007 | 0.544 | 0.511 |
| Assay Plate 2 | Calcium Ionophore A23187 | Run#1 | 4.146 | 4.440 | 12.910 | 10.980 | 8.004 | 8.210 |
|  |  | Run#2 | 2.787 | 2.273 | 4.002 | 4.308 | 5.193 | 5.261 |
|  | Beta Lapachone | Run#1 | 2.034 | 2.250 | 3.008 | 2.712 | 3.009 | 3.171 |
|  |  | Run#2 | 1.763 | 1.606 | 2.702 | 2.371 | 2.393 | 2.891 |
|  | Voriconazole | Run#1 | 0.792 | 0.113 | 0.130 | 0.114 | 0.083 | 0.083 |
|  |  | Run#2 | 0.116 | 0.094 | 0.140 | 0.122 | 0.071 | 0.094 |
|  | Sanguinarine chloride hydrate | Run#1 | 3.762 | 6.369 | 5.944 | 6.316 | 5.510 | 5.104 |
|  |  | Run#2 | 8.126 | 4.997 | 5.781 | 7.068 | 5.115 | 5.853 |
|  | PD-166285 hydrate | Run#1 | >50 | >50 | >50 | 29.460 | 11.960 | 8.225 |
|  |  | Run#2 | >50 | >50 | >50 | 47.370 | 21.970 | 18.760 |
|  | Eliprodil | Run#1 | >50 | >50 | >50 | 26.870 | 11.440 | 10.740 |
|  |  | Run#2 | >50 | >50 | >50 | 27.450 | 13.280 | 16.880 |
|  | U73343 | Run#1 | >50 | >50 | >50 | >50 | >50 | >50 |
|  |  | Run#2 | >50 | >50 | >50 | >50 | >50 | >50 |
|  | AS 604850 | Run#1 | 6.927 | 7.625 | 7.014 | 6.036 | 3.967 | 4.120 |
|  |  | Run#2 | 4.472 | 2.602 | 5.219 | 4.863 | 3.460 | 4.405 |
|  | Indirubin-3'-oxime | Run#1 | >50 | >50 | >50 | >50 | 31.750 | 16.650 |
|  |  | Run#2 | >50 | >50 | >50 | >50 | 15.270 | 20.410 |
|  | Tyrphostin AG 879 | Run#1 | 4.702 | 8.225 | 15.200 | 30.830 | 16.710 | 15.190 |
|  |  | Run#2 | 3.388 | 3.245 | 7.455 | 8.167 | 6.946 | 9.802 |
|  | TBBz | Run#1 | 4.374 | 10.270 | 8.067 | 8.501 | 5.333 | 4.937 |
|  |  | Run#2 | 7.440 | 3.979 | 5.426 | 6.302 | 4.384 | 5.046 |
|  | SR 59230A | Run#1 | >50 | 44.300 | 29.070 | 25.880 | 17.750 | 14.820 |
|  |  | Run#2 | >50 | 37.830 | 31.720 | 24.160 | 14.680 | 15.400 |
|  | SU 5416 | Run#1 | >50 | 0.416 | 3.107 | 2.688 | 2.011 | 1.974 |
|  |  | Run#2 | >50 | 1.022 | 2.465 | 0.744 | 1.206 | 2.522 |
|  | Artemether | Run#1 | >50 | 1.473 | 15.420 | 19.900 | 19.580 | 11.920 |
|  |  | Run#2 | >50 | 4.112 | 11.760 | 11.020 | 6.279 | 15.380 |
|  | LP 12 hydrochloride hydrate | Run#1 | 7.127 | 20.550 | 15.480 | 14.830 | 9.261 | 7.671 |
|  |  | Run#2 | 20.090 | 16.780 | 17.550 | 18.950 | 10.120 | 12.680 |
| Assay Plate 3 | CGP 57380 | Run#1 | >50 | >50 | >50 | >50 | >50 | >50 |
|  |  | Run#2 | >50 | >50 | >50 | >50 | >50 | >50 |
|  | Auranofin | Run#1 | 3.154 | 3.849 | 4.615 | 5.434 | 4.304 | 4.615 |
|  |  | Run#2 | 1.517 | 1.715 | 3.114 | 2.799 | 3.227 | 3.973 |
|  | Indatraline hydrochloride | Run#1 | 23.650 | 19.320 | 14.740 | 12.950 | 11.190 | 12.240 |
|  |  | Run#2 | 38.630 | 20.920 | 22.770 | 20.870 | 14.780 | 15.400 |
|  | Bay 11-7085 | Run#1 | 1.774 | 2.476 | 4.186 | 6.561 | 5.130 | 5.543 |
|  |  | Run#2 | 0.365 | 0.524 | 0.986 | 1.029 | 1.284 | 1.638 |
|  | BIO | Run#1 | 5.453 | 7.281 | 17.830 | 22.550 | 14.420 | 17.020 |
|  |  | Run#2 | 2.778 | 3.714 | 13.610 | 16.730 | 10.750 | 19.100 |
|  | Sertraline hydrochloride | Run#1 | 24.050 | 20.510 | 15.560 | 16.760 | 12.380 | 14.810 |
|  |  | Run#2 | 26.420 | 23.490 | 32.090 | 26.980 | 19.850 | 25.210 |
|  | Chelerythrine chloride | Run#1 | >50 | >50 | >50 | >50 | >50 | >50 |
|  |  | Run#2 | >50 | >50 | >50 | >50 | >50 | >50 |
|  | GBR 12935 dihydrochloride | Run#1 | 33.600 | 29.390 | 19.150 | 17.790 | 9.846 | 9.188 |
|  |  | Run#2 | 26.720 | 21.560 | 22.650 | 23.530 | 9.005 | 11.210 |
|  | AC 93253 iodide | Run#1 | 3.663 | 5.015 | 4.801 | 5.300 | 3.774 | 3.515 |
|  |  | Run#2 | 1.635 | 1.877 | 4.081 | 4.608 | 3.298 | 4.435 |
|  | Calmidazolium Chloride | Run#1 | 9.033 | 6.817 | 6.864 | 7.999 | 6.043 | 5.733 |
|  |  | Run#2 | 4.045 | 4.439 | 4.602 | 4.369 | 4.358 | 4.354 |
|  | IMS2186 | Run#1 | >50 | >50 | >50 | >50 | >50 | >50 |
|  |  | Run#2 | >50 | >50 | >50 | >50 | >50 | >50 |
|  | Nocodazole | Run#1 | >50 | 2.284 | 1.108 | 1.505 | 0.802 | 0.784 |
|  |  | Run#2 | >50 | 0.951 | 0.930 | 0.788 | 0.530 | 0.519 |
|  | PD-173952 | Run#1 | >50 | >50 | 25.240 | 22.520 | 4.124 | 3.398 |
|  |  | Run#2 | >50 | >50 | 22.140 | 20.190 | 3.579 | 6.756 |
|  | Tyrphostin A9 | Run#1 | 0.840 | 1.136 | 1.555 | 2.480 | 1.912 | 2.508 |
|  |  | Run#2 | 0.198 | 0.302 | 1.066 | 1.135 | 1.035 | 2.129 |
|  | Stattic | Run#1 | 3.742 | 4.391 | 5.410 | 6.797 | 6.422 | 6.181 |
|  |  | Run#2 | 2.392 | 2.059 | 4.054 | 4.439 | 4.803 | 6.365 |
|  | Methiothepin mesylate salt | Run#1 | 25.400 | 21.070 | 14.800 | 12.900 | 10.850 | 11.890 |
|  |  | Run#2 | 29.520 | 20.840 | 22.770 | 22.200 | 15.100 | 16.250 |

Supplemental Table 2. IC_50_ values for each compound and time point over the 120-hour experiment.

| Supplemental Table 3. Breakdown of treatment groups | | | | | | | |
| --- | --- | --- | --- | --- | --- | --- | --- |
| Experiment | Drug | # of groups | Group | Treatment | | Dose | |
| 1 | Sertraline | 4 | 1 | PBS | | 20 | mL/kg/day |
|  |  |  | 2 | Sertraline | | 15 | mg/kg/day |
|  |  |  | 3 | Fluconazole | | 15 | mg/kg/day |
|  |  |  | 4 | combination therapy with | Sertraline | 15 | mg/kg/day |
|  |  |  |  |  | Fluconazole | 15 | mg/kg/day |
| 2 | Tamoxifen | 4 | 1 | peanut oil | |  |  |
|  |  |  | 2 | Tamoxifen | | 200 | mg/kg/day |
|  |  |  | 3 | Fluconazole | | 15 | mg/kg/day |
|  |  |  | 4 | combination therapy with | Tamoxifen | 200 | mg/kg/day |
|  |  |  |  |  | Fluconazole | 15 | mg/kg/day |
| 3 | Vanoxerine | 4 | 1 | PBS | |  |  |
|  |  |  | 2 | Vanoxerine | | 10 | mg/kg/day |
|  |  |  | 3 | Fluconazole | | 15 | mg/kg/day |
|  |  |  | 4 | combination therapy with | Vanoxerine | 10 | mg/kg/day |
|  |  |  |  |  | Fluconazole | 15 | mg/kg/day |

Supplemental Table 3. Breakdown of treatment groups. The name of the drug, dosage and number of mice used in each group. The sertraline and tamoxifen experiments occurred simultaneously with 10 mice in each group. Five additional fluconazole only mice were included in the vanoxerine study, increasing the fluconazole controls to 15 mice total. Only 4 PBS control mice and 5 untreated infections were included in each experiment.
